# The neural basis of meta-volition

**DOI:** 10.1038/s42003-019-0346-1

**Published:** 2019-03-14

**Authors:** Parashkev Nachev, R. Edward Roberts, Masud Husain, Christopher Kennard

**Affiliations:** 1 Institute of Neurology, UCL, 33 Queen Square, London, WC1N 3BG UK; 2 Faculty of Medicine, Imperial College London, St Dunstan’s Road, London, W6 8RP UK; 3 Department of Clinical Neurology, University of Oxford, Level 6, West Wing, John Radcliffe Hospital, Headley Way, Oxford, OX3 9DU UK

## Abstract

Volition is the power to act beyond simple, automatic responses. We can act voluntarily because we can choose to act otherwise than immediate, external circumstances dictate. But we can also choose to allow ourselves to be led automatically by events around us. The neural basis of this higher power to suspend volition— which we term meta-volition—is unknown. Here we show that inter-individual differences in meta-volition are reflected in extensive, highly lateralised differences in right frontal white matter as indexed by diffusion tensor imaging. Paradoxically, participants with enhanced white matter optimality in these regions are less able to exercise meta-volition, finding it harder to suspend volition. This suggests volition is dependent less on any hierarchical system of meta-volitional control than on the extent to which an extensive network subserving higher volitional powers is competitively dominant over others. A fundamentally parallel neural organisation of human voluntary action at the highest level is thereby implied.

## Abstract (web summary)

Parashkev Nachev et al. investigate the neural basis of meta-volition, the ability to choose to suspend volition. Using diffusion tensor imaging, they find that less optimized right frontal white matter was correlated with improved meta-volition abilities, suggesting meta-volition is controlled by a parallel rather than hierarchical neural organization.

## Introduction

The power to act voluntarily is volition: the ability to choose one’s actions^1,2^. But choice does not stop there. One also has the power to choose not to choose, deliberately to allow one’s actions to be determined automatically by surrounding events. Though other reflexive cognitive processes are the focus of intense attention^3,4^, such “volition-on-volition”—meta-volition, for short—has not been previously investigated. Yet the nature of its neural instantiation is of crucial importance to the fundamental organisation of voluntary action because meta-volition necessarily lies hierarchically higher than volition. Indeed, it definitionally lies at the apex of any system of voluntary action control.

This crucial position of meta-volition allows us to examine a key question. Within the domain of human voluntary action, the neural organisation of the brain exhibits structural features suggestive of both serial or “hierarchical”, and parallel or “democratic” modes of operation^5–7^. The extent to which one or the other predominates is often difficult to determine, for the organisation is generally complex enough to allow either type of model to be fitted to the data comparably well. There are nonetheless critical points where relatively sharp constraints on the space of plausible models may be found. Within any hierarchical model, such a critical point is the apex, the highest node to which all other nodes are necessarily subjugated. A predominantly hierarchical neural organisation of volition would therefore predict the existence of a distinct neural substrate for meta-volition, explicitly governing the operation of volitional powers.

Meta-volition, however, can also be instantiated by a fundamentally parallel class of models, where the power to suspend volition and to respond involuntarily is simply the outcome of democratic competition between the neural substrates of actions differing in their degree of voluntariness, including automatic, involuntary actions^5–8^. Here meta-volition emerges as a purely latent variable, without a distinct neural substrate. A predominantly parallel neural organisation of volition would therefore predict meta-volition to be determined largely by the competitive balance between neural substrates across the range of action—from fully voluntary to fully automatic—not by the operation of an explicit meta-volitional node.

These two approaches to modelling volition make divergent predictions about inter-individual variations in the neural substrate of meta-volition. In the serial case, greater powers of meta-volition across individuals ought to be reflected in enhanced optimality of a dedicated meta-volitional substrate. In contrast, in the parallel case, such greater powers ought to be reflected in reduced optimality of the substrates of more voluntary actions compared with less voluntary ones, allowing the automatic competitively to overcome the voluntary more easily. A behavioural index of meta-volition combined with a neuroanatomical tool sensitive to functionally material structural brain differences across individuals offers a direct test of these divergent possibilities. Making this distinction sets a limit to the maximal level of the hierarchy of any serial model of volition.

Behavioural paradigms designed to study voluntary action usually require the suppression of a less voluntary, automatic action so as to perform a more voluntary, deliberate one. To derive a behavioural measure of meta-volition we need to do the opposite: respond automatically within a task where the inclination to respond deliberately arises as interference, degrading rather than improving performance. The meta-volitional power to suppress this inclination—to withhold volition—is then directly measured by the degree of interference on the automatic response. For a behavioural task to yield such a measure it must have two key features. First, optimal performance must depend solely on an external stimulus that guides the behaviour directly. Second, there must be nothing voluntary that one can do so as to improve performance other than to allow the external stimulus to be guiding. Any specific voluntary contribution to the action can therefore only be counterproductive overall. This second requirement is the hardest to achieve, for the only reliable way to stop the subject from attempting to do something task specific is to render the critical task specifics unconscious.

**Fig. 1:**
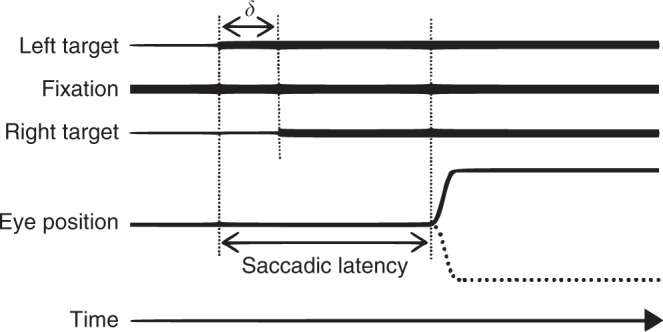
Diagrammatic sketch of the asynchronous saccadic choice paradigm. Participants viewed a horizontally arranged array of three targets where the central target served as the fixation point. On each trial, the participant made a single speeded saccade in response to the sudden illumination of one or both of the peripheral targets. On single target trials (50%) only one randomised peripheral target was illuminated and the subject made a saccade to it. On double target trials, both peripheral targets were illuminated but with a small asynchrony (*δ*) between them, and the participant was instructed to foveate as rapidly as possible whichever target caught his attention. Critically, participants were not instructed to choose the target that they consciously perceived to have occurred first, but to allow their gaze to shift automatically to whichever target caught their attention, unconsciously guided by the bias afforded by the asynchrony. The value of *δ* on each double saccade trial was selected by an automatic adaptive algorithm that optimised the information gain about the underlying function^18^. Participants performed 400 trials in total after a training run of 50 trials which was not used in the analysis. Details of the performance of each participant are given in Fig. 3. See Methods for further details

**Fig. 2:**
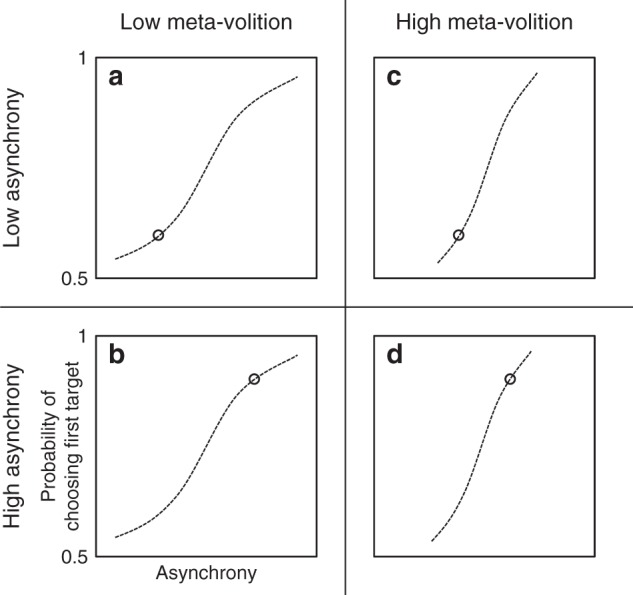
Measuring meta-volition with asynchronous choice. The relation between the probability of choosing the first target and the target asynchrony (*δ*) is a monotonically increasing function ranging from chance to 1. When *δ* is low the probability of choosing the first target is closer to chance (plot **a**) than when *δ* is high (plot **b**). The degree to which *δ* determines the response is modulated by the degree of interference of factors other than *δ* such as volitional factors disposing the subject arbitrarily to look one way or the other. This modulation is reflected in the slope of the function. Subjects who are able to minimise such volitional interference, exercising greater power of meta-volition, show steep functions determined sharply by the value of *δ* (plots **c**, **d**). Subjects who are less able to minimise volitional interference, reflecting weaker powers of meta-volition, show flatter functions less sharply determined by the value of *δ* (plots **a**, **b**). The slope is thus the measure of meta-volition. See Fig. 3 for plots of the functions of each participant in the experiment

These two requirements are satisfied by the asynchronous saccadic choice paradigm^9^. Here on each trial participants are asked to make a saccade, as quickly as they can, to the sudden onset of either of two peripheral visual targets, dependent on which one catches their attention (Fig. 1). The asynchrony (*δ*) of onset between the two targets is varied within a range of small values (typically <80 ms) so as to bias responding towards one or the other target, randomised across trials. At a low *δ* the bias is weak and the probability of foveating the first target is closer to chance (Fig. 2a); at a high *δ* the bias is strong and the probability of foveating the first target is closer to 1 (Fig. 2b). The behavioural bias is thus readily parameterised as a psychophysical “choice” function—with a characteristic threshold and slope—smoothly relating the size of the asynchrony to the probability of choosing the first target. Optimally performed, the only influence on the saccadic choice in the task would be *δ*, resulting in a sharp function ranging from chance to a probability of 1 within the smallest possible value of *δ*.

Crucially, participants are instructed not to choose the target they consciously perceived to have occurred first, but rather to respond spontaneously, allowing their eyes to be automatically guided by the cues without any conscious perceptual intervention. This is possible because the asynchrony at which a consistent saccadic response bias can be achieved is typically half the threshold at which the temporal order of target onset can be consciously perceived^9,10^. The optimal choice therefore occurs beneath the threshold of conscious perception on which any deliberate, volitional choice would necessarily depend. A deliberate choice based on conscious perception of the onset would therefore hinder performance rather than help it, for at asynchronies capable of strongly biasing a spontaneous response the conscious perception of priority will be much weaker. Volitional processes here can therefore only add interference noise to the low-level, automatic bias produced by the asynchrony.

It is impossible to eliminate such interference completely. On each trial in addition to the asynchrony, other, undefined factors will influence which way the subject will go—guesses, spontaneous inclinations, etc.—necessarily more complex voluntary response tendencies than the simple *δ* bias. Critically, we do not need to know what these factors are; indeed, we cannot know for we would have to capture the full spectrum of possible voluntary patterns of responding: any factor other than *δ* can only degrade performance. This degradation is captured psychophysically by the slope of the choice function^11^. In participants in whom the volitional interference is high the slope will be flat (Fig. 2a, b), where it is low it will be steep (Fig. 2c, d). Our measure of the capacity to withhold volition, to minimise the interference from these factors, is then simply the slope of the function: the steeper the slope in each participant the better the power of meta-volition.

To examine the link between inter-individual differences in behaviour and the underlying neural architecture we chose magnetic resonance diffusion tensor imaging (DTI) of the white matter, a method increasingly shown to reveal functionally relevant neural variation with great sensitivity^12–16^. We focussed on diffusivity measures, in particular axial diffusivity, a regional marker of white matter optimality that is usually positively correlated with the performance of the powers dependent on the neural substrate. A dominance of areas showing a positive correlation with DTI indices of white matter optimality would therefore support a serial framework of volition where inter-individual differences in meta-volition are primarily driven by the optimality of an explicit meta-volitional node. Conversely, a dominance of areas showing a negative correlation—i.e. better performance in those with less optimised white matter—would support a parallel framework where such differences are primarily driven by the competitive balance between the substrates of voluntary and automatic powers, and meta-volition emerges as a purely latent variable. In these circumstances having an optimised network subserving voluntary powers reduces performance because its interference cannot be easily overcome by the networks subserving simpler, more automatic powers. Note the optimality we are referring to is only of the connective efficiency, not of the operation of the network as a whole, which is naturally not determinable from white matter parameters alone.

## Results

### Behaviour

**Fig. 3:**
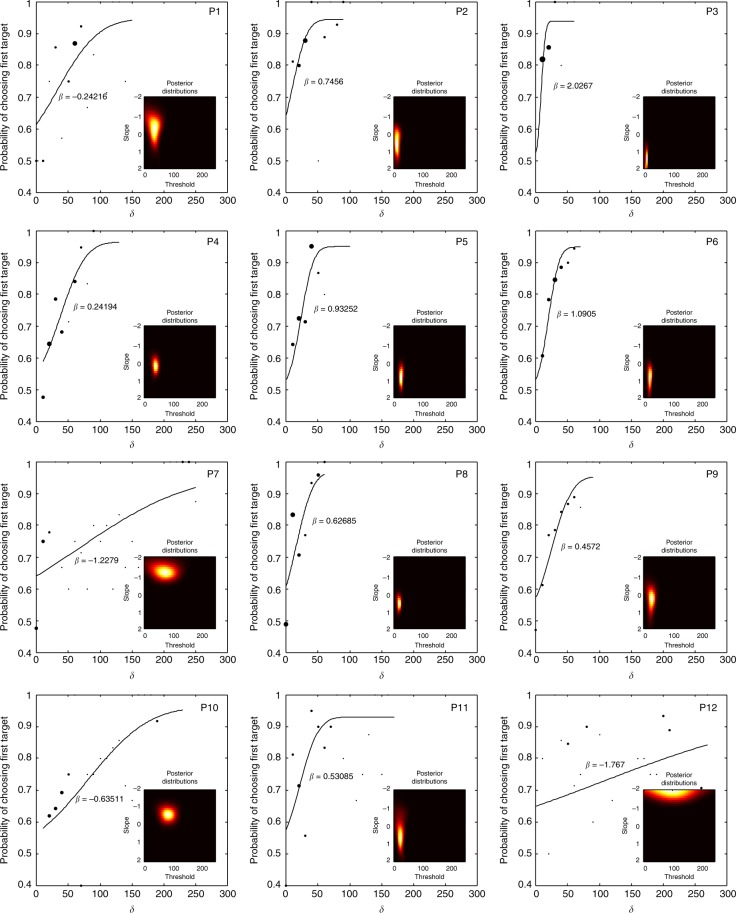
Saccadic choice function plots. On each pair of axes is plotted the final estimate of the underlying psychometric function with the slope value (*β*) indicated next to the line. The dots show the values of *δ* sampled by the algorithm, with the diameter of each dot proportional to the number of observations at that value. Note that since the algorithm adaptively sampled the function of each subject in response to individual performance, the values of *δ* necessarily vary across the group. Note also that the function is not a post hoc fit to the values of *δ* but rather is estimated adaptively during the run. The heatmaps within each plot show the densities of the posterior distributions of the slope and threshold parameters at the end of the run. Each function was estimated on the basis of 200 trials. Detailed data for the 13th participant is not available owing to accidental corruption of the digital file storing the details of the function

Any behavioural measure is only fruitfully correlated with brain structure if it shows variation across people. The 13 participants tested yielded a range of functions differing widely in their slopes (Fig. 3) indicating a range of powers of meta-volition. To estimate the individual slopes as accurately as possible we manipulated *δ* following a Bayesian adaptive algorithm that tracked performance trial-by-trial, automatically selecting the asynchrony of successive trials so as to maximise the information about the underlying psychometric function^17^. Crucially, this approach allows us to derive robust estimates of slope, including Bayesian posterior probability distributions of it for each participant (Fig. 3, insets), showing that the variation we are observing is unlikely to be simply noise within a parameter that is difficult to estimate. Bayesian methods have been shown to be optimal for accurate estimation of psychometric functions^18^.

### Imaging

**Fig. 4:**
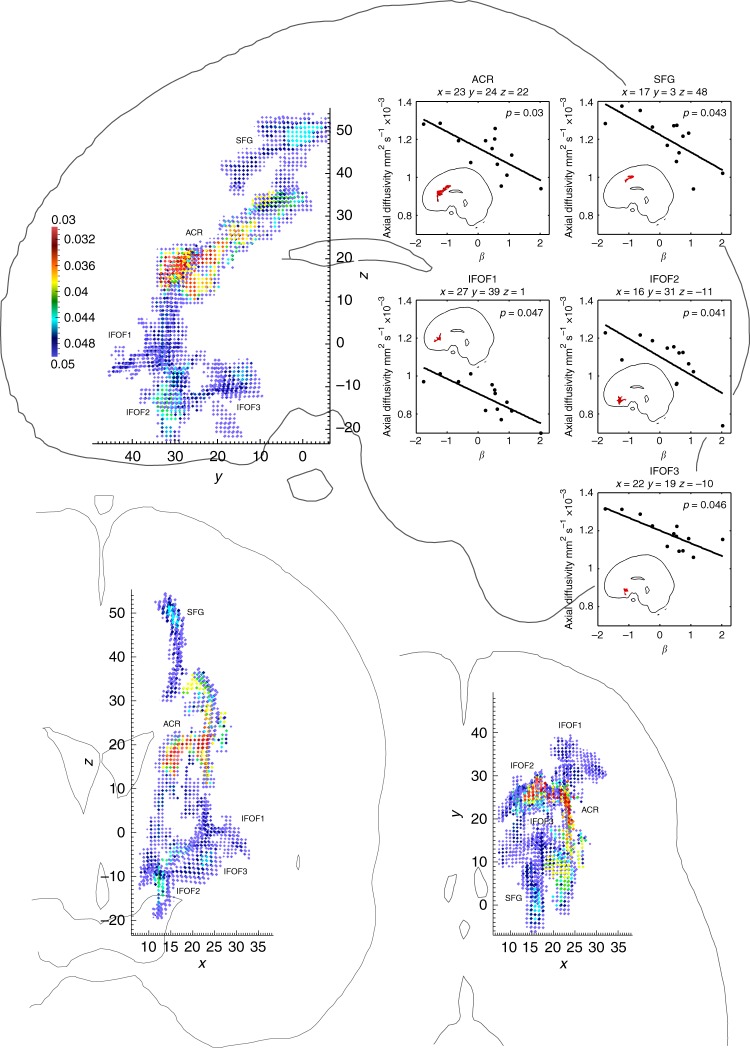
Axial diffusivity correlates of meta-volitional ability. Three-dimensional volume glyph plots showing white matter voxels with axial diffusivity significantly correlated with the slope parameter (*β*) of the choice psychometric function. Sagittal, coronal, and axial views of the five significant clusters falling within the anterior corona radiate (ACR), superior frontal gyrus white matter (SFG), and inferior fronto-occipital fasciculus (IFOF) are shown. Each plot is not a section but a volume rendering where the value of each voxel is given by a glyph coloured by the *p* value, depth being indicated by the stack of glyphs at that point. The conjunction of the three views allows each voxel to be identified unambiguously. The MNI coordinates are given for each view. The line sections are contours drawn through voxels of value 100 in the MNI 152 T1 volume, cut at *x* = 10 for the axial view, *y* = 0 for the coronal view, and *z* = 0 for the axial view. The subplots show the cluster level raw data for each sub-region, giving the corrected *p* value of the correlation in the top right corner, with a regression fit for illustration. The axial brain icons in each of these subplots indicate the extent of the corresponding cluster. Results were considered significant at *p* < 0.05 and clusters with over 100 contiguous voxels, corrected for multiple comparisons using threshold-free cluster enhancement (TFCE), an approach which avoids the choice of an arbitrary threshold for initial cluster formation. See Methods for further details. A manipulable three-dimensional rendering of the data presented here is available in Supplementary Figure 1

The individual behavioural parameters were then correlated with the imaging to identify the plausible neural substrate of the behaviour. Whole-brain DTI volumes acquired for each participant with a 1.5 Tesla scanner were used to derive individual maps of white matter diffusivity. Following nonlinear registration of the images so as to bring them into a common stereotactic space, these maps were used to construct a voxel-wise statistical model where the significance of the correlation between the slope parameter of the choice function with the diffusivity was evaluated for each location in the white tracts common to the group, controlled for age and sex, and corrected for multiple comparisons. This analysis revealed an extensive, highly lateralised region connecting the right frontal and prefrontal cortex, showing strongly significant negative correlation of axial diffusivity with the meta-volitional measure (Fig. 4). The region consisted of five contiguous clusters: one falling within the right anterior corona radiata, one underlying the right superior frontal gyrus, and three falling within the right inferior fronto-occipital fasciculus (according to the John Hopkins University white matter tractography atlas^19^). The cortex overlying these lateralised regions is heavily implicated in complex voluntary action, with a lateralisation in the literature that is congruent with our findings here^20–22^. These suggesting that the strength of connectivity of these areas weakens the power of meta-volition as a consequence of greater interference from volitional substrates. By contrast, no areas were found to be positively correlated with meta-volitional power beyond the threshold of significance, or with measures of radial diffusivity.

## Discussion

The surprising extent and distribution of the areas identified here makes it highly improbable that they are critical to any single function, still less meta-volition, with the behavioural parameters of which they show in any event an inverse correlation. So broad a swathe of overlying cortex must underpin a wide range of voluntary action possibilities, consistent with our model of interference in the task where any potential voluntary action plan may cause interference. Equally, the striking lateralisation and confinement within the frontal lobe make the result unlikely to reflect a global, functionally non-specific difference in the white matter that happens incidentally to correlate with the index of meta-volition.

Nonetheless, we must consider alternative explanations. Though the task necessarily involves a perceptual component—detecting the priority of onset of the two cues—no perceptual process can plausibly account for the observed correlation for five reasons. First, correlating the threshold of the function—a better measure of perceptual sensitivity than slope—with the imaging parameters yielded no surviving voxels for either a positive or a negative correlation with either axial or radial diffusivity. Second, these frontal areas are remote from the posterior areas known to be principally concerned with visual perception. Third, optimal performance of the task is below the threshold of conscious perception^9^. Fourth, the observed confinement to the frontal lobe is inconsistent with any localisation that might be expected from decisive involvement of an attentional network^23^. Fifth, white matter optimality is here inversely correlated with performance.

This result is also not plausibly explained by simple motor aspects of saccade generation. The critical areas of nearby cortex for saccades—the frontal and supplementary eye fields—are both bilateral and far more circumscribed than the extensive white matter differences would suggest^24^. The low level, collicular circuits likely to be dominantly engaged by the task, automatically performed, are of course wholly out of range, being subcortical. Enhanced optimality of the white matter connections of these substrates would in any event predict improved performance rather than the inverse relation observed here.

Finally, behaviour within the scanner*—*how motionless the participants were during image acquisition—could cause artefactual correlations owing to attenuation of measured diffusivity from incidentally greater head movement of participants who were worse at the behavioural task. Such an effect could not explain our result, however, because any movement-induced false correlations would either be global or distributed on either side of a common axis of head movement: clearly this cannot be true of an isolated right frontal localisation.

In the absence of alternative explanations we are compelled to conclude that the strong inverse relationship between white matter optimality in regions serving right frontal cortex and the power of meta-volition indicates that the highest level of control of voluntary action is mediated primarily by a competitive process between parallel systems subserving actions of varying levels of voluntariness rather than a hierarchically superior “meta-volitional system”. The idea of action selection as the outcome of democratic competition between rival neural ensembles—as elegantly conceptualised in the LATER model^25^ and its kin—therefore appears to extend to the highest level of volition. This empirical finding substantiates the conceptual implausibility of models of action where decision-making is localised to hierarchically ever higher substrates, for such models both needlessly limit the size of the substrate on which volitional powers are dependent and fundamentally displace rather than answer the question of how the brain makes the exercise of volitional powers possible. It offers support instead to democratic models of neural organisation^5^ where volition—including the highest power of suspending volition—arises competitively without the intervention of a discrete hierarchical system of higher control.

## Methods

### Participants

A group of 13 right-handed participants aged 21–28 (mean 22.9) with a sex ratio of 6:7 M:F took part in the study. The behavioural task and the imaging were administered in separate sessions. Informed written consent was obtained, and the study was approved by the London–West London & GTAC Research Ethics Committee.

### Behavioural task

Participants were seated in front of a cathode ray tube screen running at 100 Hz vertical refresh rate (thereby allowing 10 ms temporal resolution between events) with their heads supported by a table-mounted chin rest while their eye position was continuously monitored by an ASL model 504 high-speed, pan-tilt, infrared video-based eye tracker (Applied Science Laboratories, Bedford, MA), sampling at 240 Hz with an ASL model 5000 series controller. They viewed a horizontally arranged array of three 0.5° targets 8° apart with the central target serving as the fixation point. No-one but the participant was in the testing room during data collection. On each of a total of 400 trials, the participant was instructed rapidly to make a single saccade in response to the sudden illumination of one or both of the peripheral targets. On single target trials (50% of the total) only one randomly chosen peripheral target was illuminated and the participant made a saccade to it. On double target trials, both peripheral targets were illuminated but with a small asynchrony (*δ*) between them, and the participant was instructed to foveate as rapidly as possible whichever target caught his attention. Critically, participants were instructed not to choose the target that they consciously perceived to have occurred first, but to allow their gaze to shift automatically to whichever target caught their attention. The value of *δ* on each double saccade trial was selected by an automatic algorithm that chose within the range 0–300 ms in 10 ms steps according to a Bayesian model of the underlying psychometric function with four parameters—guess rate (fixed at 0.5), threshold (0–300 ms), slope (−2 to 2), and lapse rate (0 to 0.1)—and logistic form. Starting with flat priors the model updated the posterior distributions of each parameter in response to performance at the given value of *δ* (starting at 300 ms) on each trial and chose the value of *δ* for the next trial so as to optimise the information gain about the underlying function^10^. For each participant, this yielded at the end of the run estimates of the threshold and slope, together with their posterior distributions. The algorithm was implemented in Matlab (Mathworks, USA) using a Bayesian adaptive psychometric methods toolbox developed by Thomas Tanner (https://www.is.mpg.de/publications/3256). Participants performed 400 trials in total after a training run of 50 trials which was not used in the analysis. Details of the performance of each participant are given in Fig. 3.

### Imaging

DTI data were acquired using a 1.5 T Siemens Magnetom Vision system at Charing Cross Hospital, London. Four sets of whole-brain diffusion-weighted volumes were acquired for each participant (12 directions; *b* = 1000 s mm^−2^; 48 slices; voxel size 2 × 2 × 3 mm^3^; repetition time (TR) = 8.6 s; echo time (TE) = 94 ms; single shell; ungated) plus four volumes without diffusion weighting (*b* = 0 s mm^−2^). A T1-weighted anatomical image was also acquired using a MP-RAGE sequence (TR = 1160 ms; TE = 4.38 ms; flip angle = 15; voxel size 1 × 1 × 1 mm^3^).

### Statistical analysis

Voxel-wise statistical analysis of the diffusion-weighted data was performed using tract-based spatial statistics, part of FSL^26,27^. Diffusivity images were created by fitting a tensor model to the raw diffusion data using FMRIB’s diffusion toolbox (FDT), and then brain extracted using Brain Extraction Tool (BET)^28^. The data were then aligned into a common space (Montreal Neurological Institute) using the nonlinear registration tool FNIRT with the MNI152 template. A mean diffusivity image was created and thinned to generate a mean diffusivity skeleton representing the centres of all tracts common to the group, within which further voxel-wise operations were confined. We applied a general linear model and used permutation-based non-parametric testing with variance smoothing and age and sex as a covariate of no interest^29^. Results were considered significant at *p* < 0.05 and clusters with over 100 contiguous voxels, corrected for multiple comparisons using threshold-free cluster enhancement, an approach that avoids the choice of an arbitrary threshold for initial cluster formation^30^. No data were excluded. The null hypothesis is about the relation between brain structure and psychophysical performance, about which the experimenter can only be blind.

### Code availability

The analysis code is available on reasonable request from the corresponding author.

### Reporting Summary

Further information on experimental design is available in the Nature Research Reporting Summary linked to this article.

## Supplementary information

Supplementary Information

Reporting Summary

## Data Availability

The processed datasets are available on reasonable request from the corresponding author.
