# Supplementary material for: The neural basis of meta-volition
Source: Commun Biol. 2019 Mar 14;2:101. doi: 10.1038/s42003-019-0346-1 (PMC6418118; doi:10.1038/s42003-019-0346-1)
Supplement: Supplementary file 1 — Supplementary Information [file 42003_2019_346_MOESM1_ESM.pdf]

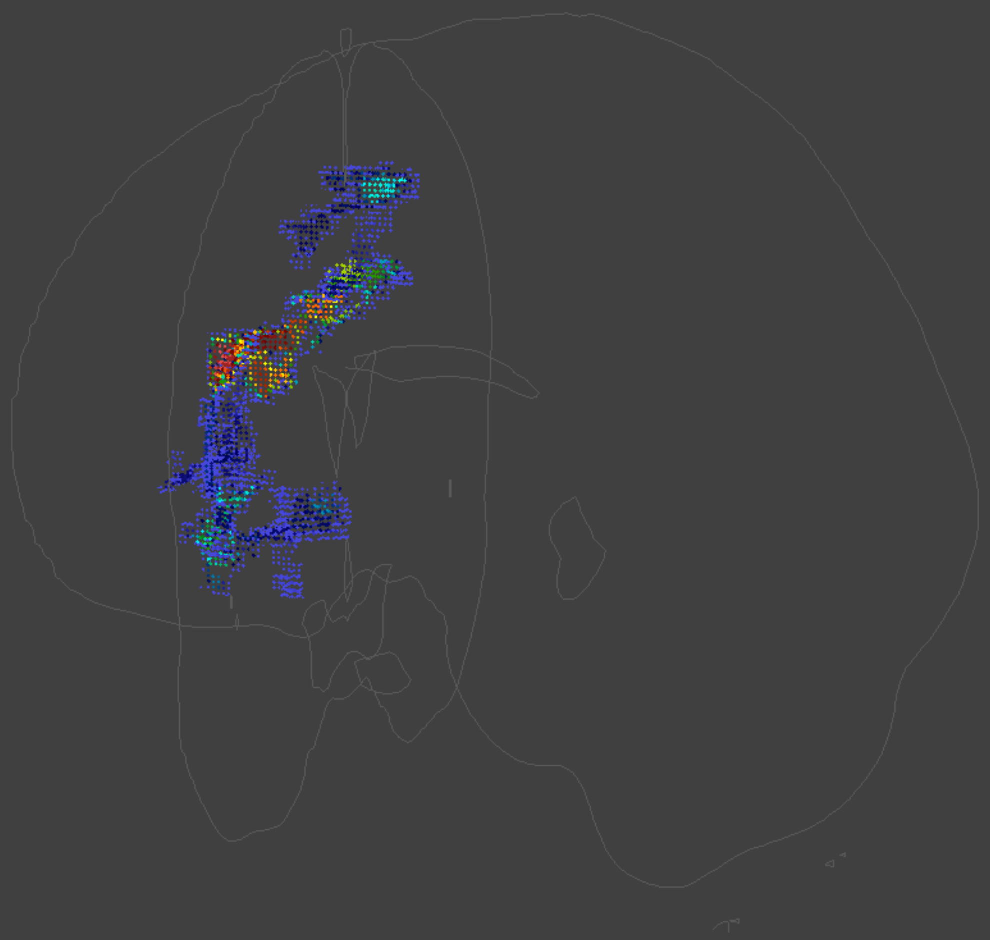

Supplementary Figure 1. Manipulable three-dimensional rendering to be viewed in Adobe Reader of the data presented in Figure 4 of the main text. The coloured glyphs show areas of white matter where the axial diffusivity is inversely correlated with the slope parameter across the group of participants at or below the threshold of significance, corrected for multiple comparisons. The black lines are cross sections of the MNI 152 T1 template at $z = 0$ for the axial plane, $y = 0$ for the coronal plane, and $x = 10$ for the sagittal plane, contoured at a voxel value of 100.
