# Supplementary material for: The neural basis of meta-volition
Source: Commun Biol. 2019 Mar 14;2:101. doi: 10.1038/s42003-019-0346-1 (PMC6418118; doi:10.1038/s42003-019-0346-1)
Supplement: Supplementary file 2 — Reporting Summary [file 42003_2019_346_MOESM2_ESM.pdf]

nature research

Corresponding author(s): Parashkev Nachev

Reporting Summary

Nature Research wishes to improve the reproducibility of the work that we publish. This form provides structure for consistency and transparency in reporting. For further information on Nature Research policies, see Authors & Referees and the Editorial Policy Checklist.

Statistical parameters

When statistical analyses are reported, confirm that the following items are present in the relevant location (e.g. figure legend, table legend, main text, or Methods section).

n/a	Confirmed	
☐	☒	The exact sample size (*n*) for each experimental group/condition, given as a discrete number and unit of measurement
☐	☒	An indication of whether measurements were taken from distinct samples or whether the same sample was measured repeatedly
☐	☒	The statistical test(s) used AND whether they are one- or two-sided<br>*Only common tests should be described solely by name; describe more complex techniques in the Methods section.*
☐	☒	A description of all covariates tested
☐	☒	A description of any assumptions or corrections, such as tests of normality and adjustment for multiple comparisons
☐	☒	A full description of the statistics including central tendency (e.g. means) or other basic estimates (e.g. regression coefficient) AND variation (e.g. standard deviation) or associated estimates of uncertainty (e.g. confidence intervals)
☐	☒	For null hypothesis testing, the test statistic (e.g. *F*, *t*, *r*) with confidence intervals, effect sizes, degrees of freedom and *P* value noted<br>*Give P values as exact values whenever suitable.*
☐	☒	For Bayesian analysis, information on the choice of priors and Markov chain Monte Carlo settings
☒	☐	For hierarchical and complex designs, identification of the appropriate level for tests and full reporting of outcomes
☒	☐	Estimates of effect sizes (e.g. Cohen's *d*, Pearson's *r*), indicating how they were calculated
☒	☐	Clearly defined error bars<br>*State explicitly what error bars represent (e.g. SD, SE, CI)*

Our web collection on statistics for biologists may be useful.

Software and code

Policy information about availability of computer code

Data collection	Neurobehavioural Systems Presentation Software (https://www.neurobs.com/)<br>Adaptive psychometric function toolbox v0.4 by Thomas Tanner (https://www.is.mpg.de/publications/3256)
Data analysis	Matlab 6.5.1<br>Adaptive psychometric function toolbox v0.4 by Thomas Tanner (https://www.is.mpg.de/publications/3256)

For manuscripts utilizing custom algorithms or software that are central to the research but not yet described in published literature, software must be made available to editors/reviewers upon request. We strongly encourage code deposition in a community repository (e.g. GitHub). See the Nature Research guidelines for submitting code & software for further information.

Data

Policy information about availability of data

All manuscripts must include a data availability statement. This statement should provide the following information, where applicable:

- Accession codes, unique identifiers, or web links for publicly available datasets
- A list of figures that have associated raw data
- A description of any restrictions on data availability

The datasets generated during and/or analysed during the current study are available from the corresponding author on reasonable request.

Field-specific reporting

Please select the best fit for your research. If you are not sure, read the appropriate sections before making your selection.

☐ Life sciences ☒ Behavioural & social sciences ☐ Ecological, evolutionary & environmental sciences

For a reference copy of the document with all sections, see nature.com/authors/policies/ReportingSummary-flat.pdf

Behavioural & social sciences study design

All studies must disclose on these points even when the disclosure is negative.

Study description	Quantiative experimental study combining structural brain imaging with psychophysical measurements.
Research sample	A group of 13 right-handed Imperial graduate and post-graduate students aged 21 to 28 (mean 22.9) with a sex ratio of 6:7 M:F took part in the study.
Sampling strategy	The number of participants was informed by consensus practice in the literature of studies combining psychophysics with structural brain imaging. All participants were randomly selected.
Data collection	Participants were seated in front of a cathode ray tube screen running at 100Hz vertical refresh rate, with their heads supported by a table-mounted chin rest while their eye position was continuously monitored by an ASL model 504 high-speed, pan-tilt, infrared video-based eye tracker (Applied Science Laboratories, Bedford, MA), sampling at 240 Hz with an ASL model 5000 series controller. They viewed a horizontally arranged array of three 0.5 degree targets 8 degrees apart with the central target serving as the fixation point. No-one but the participant was in the testing room during data collection. The null hypothesis is about the relation between brain structure and psychophysical performance, about which the experimenter can only be blind.
Timing	5th November 2005 to 31st January 2006
Data exclusions	No data were excluded.
Non-participation	None.
Randomization	There were no separate groups.

Reporting for specific materials, systems and methods

Materials & experimental systems

n/a	Involved in the study	
☒	☐	Unique biological materials
☒	☐	Antibodies
☒	☐	Eukaryotic cell lines
☒	☐	Palaeontology
☒	☐	Animals and other organisms
☐	☒	Human research participants

Methods

n/a	Involved in the study	
☒	☐	ChIP-seq
☒	☐	Flow cytometry
☐	☒	MRI-based neuroimaging

Human research participants

Policy information about studies involving human research participants

Population characteristics	See above.
Recruitment	By ethically-approved advert within internal university channels.

Magnetic resonance imaging

Experimental design

Design type	Single time point structural and diffusion imaging

Design specifications | One imaging session per participant.

Behavioral performance measures | Adaptively sampled oculomotor psychometric function as detailed in the manuscript.

Acquisition

Imaging type(s) | Structural and diffusion

Field strength | 1.5T

Sequence & imaging parameters | DTI: 12 directions; b=1000smm-2; 48 slices; voxel size 2x2x3mm3; repetition time (TR) = 8.6s; echo time (TE) = 94ms), plus four volumes without diffusion weighting (b=0smm-2).

Structural: T1-weighted anatomical image was also acquired using a MP-RAGE sequence (TR=1160ms; TE=4.38ms; flip angle=15; voxel size 1x1x1mm3).

Area of acquisition | Whole brain

Diffusion MRI ☒ Used ☐ Not used

Parameters | 12 directions; b=1000smm-2; single shell, ungated.

Preprocessing

Preprocessing software | Voxelwise statistical analysis of the diffusion-weighted data was performed using tract-based spatial statistics, part of FSL. Diffusivity images were created by fitting a tensor model to the raw diffusion data using FMRIB's diffusion toolbox (FDT), and then brain-extracted using Brain Extraction Tool (BET).

Normalization | The data were then aligned into a common space using the nonlinear registration tool FNIRT.

Normalization template | MNI152

Noise and artifact removal | None

Volume censoring | As implied from above.

Statistical modeling & inference

Model type and settings | We applied a mass univariate general linear model and used permutation-based non-parametric testing with variance smoothing and age and sex as a covariate of no interest.

Effect(s) tested | Correlation between axial diffusivity and the slope parameter of the psychometric function.

Specify type of analysis: ☐ Whole brain ☒ ROI-based ☐ Both

Anatomical location(s) | FMRIB58_FA-derived white matter skeleton.

Statistic type for inference (See Eklund et al. 2016) | Results were considered significant at p<0.05 and clusters with over 100 contiguous voxels.

Correction | Corrected for multiple comparisons using threshold-free cluster enhancement (TFCE), an approach which avoids the choice of an arbitrary threshold for initial cluster formation.

Models & analysis

n/a	Involved in the study	
☒	☐	Functional and/or effective connectivity
☒	☐	Graph analysis
☒	☐	Multivariate modeling or predictive analysis
